# Hyperproliferation markers in ear canal epidermis

**DOI:** 10.1590/S1808-86942010000500023

**Published:** 2015-10-22

**Authors:** João Daniel Caliman e Gurgel, Celina Siqueira Barbosa Pereira, Adriana Leal Alves, Fernando Quintanilha Ribeiro

**Affiliations:** 1 MSc and PhD student in Medicine - (Otorhinolaryngology) - School of Medical Sciences of the Santa Casa de São Paulo hospital, otorhinolaryngologist and maxillofacial surgeon; 2 PhD in Medicine (Otorhinolaryngology) - School of Medical Sciences of the Santa Casa de São Paulo hospital, Assistant Professor - Department of Morphology - School of Medical Sciences - Santa Casa de São Paulo hospital; 3 PhD in Medicine (Otorhinolaryngology) - School of Medical Sciences of the Santa Casa de São Paulo hospital, Professor - Department of Morphology - School of Medical Sciences - Santa Casa de São Paulo hospital; 4 PhD in Medicine (Otorhinolaryngology) - Paulista School of Medicine, Adjunct Professor - Department of Otorhinolaryngology - School of Medical Sciences - Santa Casa de São Paulo hospital

**Keywords:** cholesteatoma, immunohistochemistry, ear canal, keratins

## Abstract

**Abstract:**

Several studies involving immunohistochemical methods to assess external auditory canal epidermis have been performed with different objectives. With this method it is possible to assess the expression of various antigens such as cytokeratins, cytokines, and hyperproliferation markers among others.

**Aim:**

to revise, describe and analyze the knowledge generated by identifiable papers published on the worldwide literature about immunohistochemical hyperproliferation markers in normal external auditory canal epidermis.

**Materials and Methods:**

systematic review of the papers published until 2009, in indexed international journals.

**Results:**

Various antigens related to hyperproliferation were investigated by immunohistochemical methods among the included papers. The most studied ones were cytokeratin 16, Ki-67 and PCNA.

**Conclusions:**

most of the studies utilized external auditory canal epidermis as control sample to study external ear or middle ear cholesteatoma with immunohistochemical methods. There is a hyperproliferative antigen concentration, such as CK16, Ki-67 and PCNA, in the annulus tympanicus, adjacent meatus and tympanic regions, mainly in the lower areas.

## INTRODUCTION

Numerous studies involving immunohistochemical methods used to assess the epidermis of the external auditory canal (EAC) have already been carried out with the most varied objectives. By means of immunohistochemistry, initially used to diagnose tumors, it is also possible to study the mechanisms of skin migration in the external auditory canal, local immune response, the expression patterns of intermediate filaments, the presence of cytokines and other antigens, as well as the evaluation of new treatment proposals for the diseases that affect the canal^1^, ^2^, ^3^.

A deep knowledge of the immunohistochemical patterns of the external auditory canal epidermis directly associated with hyperproliferation helps understand the genesis of the many diseases which affect it (EAC cholesteatoma, benign necrotizing external otitis; epidermal packing), besides contributing to the choice of the best treatment and to establish the prognosis for each disease^3^.

One of the most widely accepted theories to explain the genesis of the middle ear acquired cholesteatoma is the external auditory canal epidermis migration to inside the tympanic cavity by means of a perforation on the tympanic membrane or by suction of its pars flacida^2^. However, there still are some controversial issues:

– Why do we have this abnormal migration in some cases when, physiologically, the local migration has a centripetal direction on the tympanic membrane and lateral on the external auditory canal?
– Why does it happen only in certain persons?
– What makes cholesteatomas more or less aggressive?

These doubts motivated the need to study the hyperproliferative characteristics of the external auditory meatus epidermis by means of immunohistochemical techniques, in the attempt to understand this peculiar behavior.

The goal of the present study was to revise, describe and analyze the expression of hyperproliferation immunohistochemical markers on the epidermis of the normal external auditory canal.

## MATERIALS AND METHODS

In order to analyze the studies involving the immunohistochemical pattern of the external auditory meatus epidermis we did a systematic review of all the identifiable papers published until the year of 2009 in internationally indexed journals about:

– Immunohistochemical methods used to study EAM epithelial samples.
– Immunohistochemical patterns of the normal EAC epidermis.
– Employment of these methods to the different diseases which affect the EAC.

In order to collect the studies we used the following electronic databases: PubMed (MEDLINE), Cochrane Collaboration Systematic Reviews (CCTR), Latin-American and Caribbean Health Literature (LILACS) and Scientific Electronic Library Online (SciELO).

For the precise choice of key-words, we analyzed the remission indexes of two periods indexed on MEDLINE with an impact factor greater than 1.0, which had papers associated with the topic in this study. They were: The Laryngoscope (impact factor in 2005: 1.617), with the paper from Vennix et al. ^[1]1^ and the International Journal of Molecular Medicine (impact factor in 2005: 2.090), with the paper from Naim et al ^[1]2^. After analyzing these papers, we chose the key-words: immunohistochemistry (IMH) and external auditory canal (EAC). We also assessed the bibliographic references of the papers selected, for the inclusion of studies which were not identified by the investigation with the chosen key-words.

The inclusion criteria were:

• Papers published in indexed journals identifiable in the world literature
• Papers directly associated to the study of immunohistochemical markers of a normal external auditory canal epidermis hyperproliferation

The exclusion criteria were:

• Identified papers without direct or indirect association with the immunohistochemical evaluation of the normal external acoustic meatus epidermis.
• Papers on the uncommon disorders of the external auditory meatus and without associations with the epithelium.
• Papers which antigens used did not have direct association with the epithelial tissue or with the cholesteatoma.

## RESULTS

The search on the electronic databases with the combination of the key-words chosen yielded 91 papers (Chart 1).

**Chart 1:** Papers found after searching the electronic databases

	IMH	EAC	IMH + EAC
PubMed (Medline)	405.268	4148	91
CCTR	808	37	0
LILACS	945	73	0
SciELO	290	15	0

PubMed: database for Medline search; Medline: Medical Literature Analysis and Retrieval System Online; CCTR: Cochrane Collaboration Systematic Reviews; LILACS: Latin American and Caribbean Literature in Health Sciences of International Literature of the Americas and the Caribbean; SciELO: Scientific Electronic Library Online; IMH: immunohistochemistry; EAC: external auditory canal.

After analyzing the selected papers, 75 were ruled out for not having direct associations with the goals of this study. With that, we only included 16 papers in this study.

As to study designs, 14 (87.5%) were cross-sectional^1^, ^2^, ^3^, ^4^, ^5^, ^6^, ^7^, ^8^, ^9^, ^10^, ^11^, ^12^, ^13^, ^14^ and only two (12.5%) were experimental^15^, ^16^.

Among all the papers included in this review, five (31.25%) were published with the only goal of analyzing, by means of immunohistochemistry, the epidermis of the normal external auditory meatus^1^,^4^,^5^,^8^,^16^. The other 11 studies (68.75%) were used only as control sample to assess the immunoexpression of cholesteatoma antigens, of the middle or external ear^2^,^7^,^8^,^10^, ^11^, ^12^, ^13^, ^14^, ^15^, ^16^.

Many markers were studied by means of the immunohistochemical methods in the investigations analyzed. Of the five papers investigating exclusively the normal EAC epidermis, we analyzed the expression of Ki-67, vimentin PCNA, IgA, IgG, IgM and many other cytokeratins^1^,^4^,^5^,^8^,^16^.

The cytokeratins were studied in four of the five studies done exclusively on the epidermis of the normal EAC as control, from a total of seven studies. The other most studied antigens were the Ki-67, in six papers and the PCNA, in three papers. (Chart 2, Chart 3).

**Chart 2:** Antigens tested exclusively on the normal external auditory meatus epidermis.

Year	Author	# of samples	Antigens tested
1993	Broekaert, Boedts	Not specified	CK 4, 5, 7, 8, 10, 13, 14, 16, 18 e 19
1993	Lepercque et al.	6	CK 4, 5, 7, 8, 10, 13, 14, 16, 18 e 19
1996	Vennix et al.	8	CK 5, 6, 7, 8, 10, 13, 14, 16, 17, 18 e 19
1997	Kakoi et al.	8	Ki-67 e PCNA
2007	Sanjuan et al.	10	CK 5, 10, 16, 17 e 19

**Chart 3:** Antigens tested on the epidermis of the external auditory canal associated with cholesteatoma

Year	Author	# of samples	Antigens tested
1992	Broekaert et al.	5	CK 4, 5, 7, 8, 10, 13, 14, 16, 18 e 19
1994	Sasaki, Huang	2	CK 13 e 16
1996	Schilling et al.	13	Ki-67
1998	Kojima et al.	10	PCNA
1998	Tanaka et al.	5	PCNA
1999	Kim, Chung	26	CK 4, 8, 10, 13, 16, 17, 18 e 19
2001	Bayazít et al.	18	P27
2003	Adamkzyc et al.	15	Ki-67
2004	Ribeiro et al.	1	CK 16 e Ki-67
2005	Raynov et al.	5	Ki-67
2006	Hwang et al.	10	Ki-67 e PPAR-gama

## DISCUSSION

Among the markers mentioned in this paper, the most studied ones were cytokeratins (CK), a complex of 20 polypeptides of the family of intermediate filaments which make up the cytoskeleton of the epithelial cells. These filaments are divided according to their molecular weight and their isoelectric point, in two subfamilies: the basic and the acid ones. One acid CK and one basic CK form a pair of CKs. Each pair can determine the differentiation stage of the keratocytes, proliferative capacity, as well as the location and environmental growth conditions. According to Ribeiro et al. ^[1]3^ CK16 is found in tissues under hyperproliferation, such as in benign hyperproliferative epidermal diseases (common wart, psoriasis, actinic keratosis and seborrheic dermatitis) and in tissues under healing stages and in malignant epidermal diseases such as squamous cell carcinoma. It is absent in the normal epidermis, except in the zones submitted to pressure and friction (feet soles, finger and heel pads) and in hair follicles coating.

The cytokeratins, despite being the more frequently studied proliferation markers, were exclusively studied in the epidermis of normal EAC only by Broekaert, Boedts ^[1]4^, Lepercque et al ^[1]5^, Vennix et al.1 and Sanjuan et al.^[1]6^. Other authors studied it in diseases of the middle and external ears, using the canal epidermis only as control. Broekaert, Boedts^4^, Lepercque et al.^5^, Vennix et al.^1^ studied the distribution of cytokeratins in the different parts of the EAC and in the outer portion of the tympanic membrane, also indicating in which epithelium layer the CK were expressed. They observed that CK16 in the most medial and inferior portion of the bony canal, in the keratocytes of the suprabasal layers. This CK was not expressed in the cartilaginous portion of the canal. According to Vennix et al.^1^, CK 16 was present in the fibrous-cartilaginous annulus, in the pars flaccida of the tympanic membrane and in its pars tensa, near the handle of malleus. Lepercque et al.^5^ noticed a gradual reduction on the intensity of the CK 16 in a centripetal direction on the membrane. This shows a proliferative capacity of the suprabasal layers of the fibrous-cartilaginous annulus. Sanjuan et al.^16^ studied the expression of cytokeratins 5, 10, 16, 17 and 19 on the epidermis of the normal EAC and in cells of the epidermal EAC from culture. The authors observed that the patterns of expression of these filaments were maintained before and after the culture, including the location of the epidermal layers seen by Lepercque et al.^5^ and Vennix et al.^1^.

**Figure 1:**
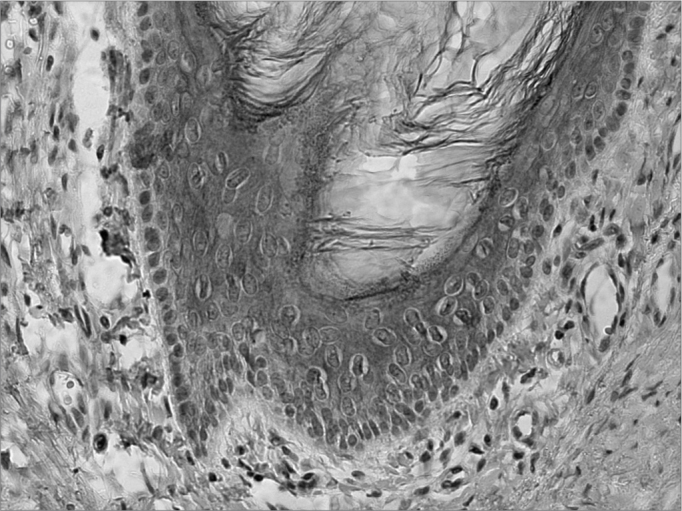
CK 16 expression on the suprabasal layers of the normal external auditory meatus

In the studies which analyzed the EAC epithelium only as control, there was CK 16 expression in the same EAC region, in the suprabasal layers; nonetheless, the authors did not report on the place from which the samples were obtained6, except for Broekaert et al.^7^ who observed CK 16 presence on the fibrocartilaginous annulus, as well as on the juxta-tympanic epithelium (Figure 1).

(source: Morphology Department - Medical Sciences School - Santa Casa de São Paulo).

**Figure 2:**
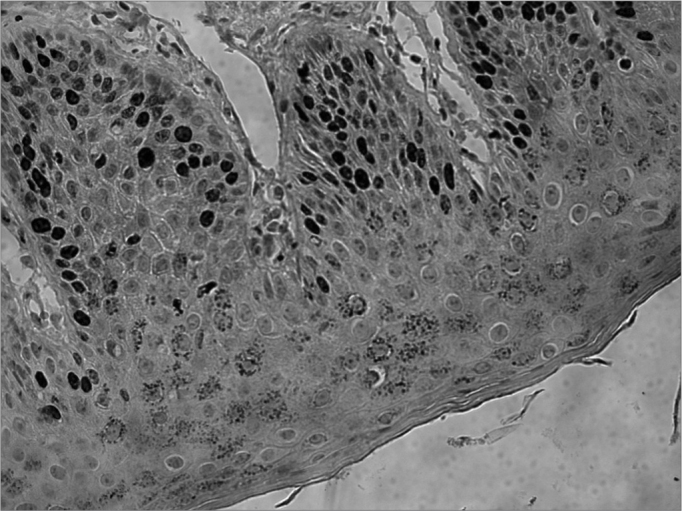
Ki-67 expression on the basal layer, extending to the suprabasal layers of the normal external auditory meatus (source: Ki-67 expression on the basal layer, extending to the suprabasal layers of the normal external auditory canal epithelium (source: Morphology Department - Medical Sciences School - Santa Casa de São Paulo).

Another very much studied marker of hyperproliferation was the Ki-67nuclear antigen, which is a marker associated with cell proliferation and was studied in five papers^8^, ^9^, ^10^, ^11^, ^12^. All the authors found expressions of this marker in the normal external auditory canal; however, only one of the papers discusses where the samples were obtained from. Kakoi et al.^8^ observed the presence of Ki-67 on the basal cells of the pars flaccida and the pars tensa of the tympanic membrane, in the fibrocartilaginous annulus and in the region near the handle of the malleus. On the canal epidermis, Ki-67 was seen, especially near the inferior annular region. Both in the EAC and in the tympanic membrane, the expression of this maker coincides with that from cytokeratin 16, showing the hyperproliferative characteristic of these regions. Schilling et al.^9^, Adamczyk et al.^10^, Raynov et al.^11^ and Hwang et al. ^[1]12^ observed a strong positiveness for Ki-67 on the EAC epidermis, without however specifying the location of where the samples were obtained from (Figure 2).

Another nuclear antigen of cell proliferation, PCNA, was studied in the normal EAC in three papers^8^,^13^,^14^. Kakoi et al.^8^, who studied this antigen together with Ki-67, found a similar pattern of expression on the basal layers of the regions near the fibrocartilaginous annulus, including the bony portion of the canal. They showed that there is a gradual reduction of its expression on the tympanic membrane in a centripetal direction, similar to the CK16. Kojima et al.^13^ and Tanaka et al.^14^ also found positiveness in the EAC epidermis PCNA, and the former observed its expression on the basal layer, extending to the suprabasal layers of the canal epidermis, and the latter only on the basal layer. Since in none of these papers there was a specific location of where the samples were taken from, it is not possible to state that only the region near the annulus is positive for PCNA.

Some other antigens associated with hyperproliferation were found on the epidermis of the external auditory canal; however, it was only in one paper and without a description of the place where the samples were taken from^12^,^15^. They may corroborate even further the hyperproliferative status of the epidermis in this region. It is the case of p27, studied by Bayazít et al.^15^ and of the PPAR-gama, by Hwang et al.^12^, which expressions were seen on the epidermis of the normal external auditory canal epidermis.

As shown by the papers hereby described, the epidermis of the external auditory canal has the expression of proliferation markers, but more specifically in its deepest part, next to the fibrocartilaginous annulus. It is in this region where we find the epithelial migration which will originate the middle ear cholesteatoma, according to a theory very much accepted for its genesis.^2^ There still is no clinical application for the routine test of these markers as a prognostic factor in patients with chronic otitis media. Nonetheless, with the progression of the studies and a greater understanding of the hyperproliferative characteristics of the EAC, many doubts will be increasingly near explanation such as, for example, the risk of developing middle ear cholesteatoma in patients with tympanic perforation, evaluation of the cholesteatoma aggressiveness or even the risk estimation of recurrence after surgical treatment.

## CONCLUSION

1 There is a concentration of hyperproliferation markers on the fibrocartilaginous annulus and on the adjacent external acoustic canal and tympanic membrane, especially on the lowest portions of the EAC;
2 CK16 if found on the normal external auditory canal in its bony portion and, on the tympanic membrane its expression reduces in the centripetal direction;
3 Ki-67 and the PCNA are also found on the bony portion of the external auditory canal and on the tympanic membrane, in the same places where we find CK 16 expression.
